# Acupuncture for reduction of opioid consumption in chronic pain: A systematic review and meta-analysis protocol

**DOI:** 10.1097/MD.0000000000018237

**Published:** 2019-12-20

**Authors:** Seunghoon Lee, Dae-Hyun Jo

**Affiliations:** a Department of Acupuncture and Moxibustion Medicine, College of Korean Medicine, Kyung Hee University; b Department of Acupuncture and Moxibustion Medicine, Graduate School, Kyung Hee University, Seoul, Republic of Korea.

**Keywords:** acupuncture, chronic pain, opioid consumption, protocol, systematic review

## Abstract

**Background:**

This review aims to evaluate the effectiveness and safety of acupuncture treatment for reducing opioid consumption in patients with chronic pain.

**Methods:**

We will search the following electronic databases from their inception to November 2019: MEDLINE, EMBASE, the Cochrane Central Register of Controlled Trials, the Cumulative Index to Nursing and Allied Health Literature, the Allied and Complementary Medicine Database, the China National Knowledge Infrastructure (a Chinese database), the Japan Science and Technology Information Aggregator (a Japanese database), and five Korean databases (KoreaMed, Research Information Service System, Korean Studies Information Service System, Database Periodical Information Academic, and Oriental Medicine Advanced Searching Integrated System). Randomized controlled trials comparing acupuncture to no treatment, sham acupuncture, and other active interventions for the reduction of opioid consumption in chronic pain patients will be included. The risk of bias will be assessed using the Cochrane risk of bias tool. The primary outcomes will include the prescribed or consumed dose of opioids and withdrawal symptoms related to opioid reduction. A meta-analysis will be performed to estimate a pooled effect, if possible.

**Conclusion:**

This study may provide important practical guidance for patients, practitioners, and health-policy makers regarding the use of acupuncture in opioid taper support programs.

**Dissemination:**

The results will be disseminated through a peer-reviewed journal or conference presentations.

Trial registration number: PROSPERO 2019: CRD42019143486

## Abstract 2

Supplemental Digital Content is available in the text

## 1 Introduction

### 1.1 Description of the condition

Chronic pain, defined as pain lasting more than 3 months, is one of the most common reasons for long-term medication prescription and affects about 20% to 50% of adults worldwide.^[1,2]^ About 8% of these individuals have high-impact chronic pain, which frequently limits life or work activities and is accompanied by various comorbidities such as anxiety, depression, and insomnia.^[2]^ As a result, the prescription of opioids has increased over the last 2 decades, especially in the United States, United Kingdom, and Australia, for patients with high-impact chronic pain as well as acute postoperate and cancer pain.^[3]^ Among the 25.3 million patients with chronic pain in the Unites States, opioids were prescribed for more than 240 million times in 2015.^[4]^ However, many studies have revealed limited evidence opioid use for long-term pain relief in chronic-pain conditions.^[5]^ Moreover, addiction and overuse of opioids leads to considerable adverse effects, such as bladder dysfunction, sedation, immune suppression, and even death.

### 1.2 Description of the intervention

Acupuncture is defined as an intervention that stimulates specific points (eg, traditional perspective acupuncture points, myofascial trigger points, or tender points) using dry needles with some kind of manipulation (eg, manual or electrical). Recently, a rigorous meta-analysis of data from 20,827 individuals showed that acupuncture is effective for nonspecific chronic musculoskeletal pain when compared to a sham control, and the treatment effects persisted over time.^[6]^ In addition to pain reduction, acupuncture treatment is a promising adjunct to facilitate the reduction or tapering of pain medications, including opioids, in patients with chronic pain.^[4]^ It may also be used to treat opioid use disorder, which may involve opioid cravings, anxiety, depression, or insomnia.^[7]^

### 1.3 How the intervention might work

Although the mechanism of acupuncture treatment for reducing opioid consumption remains unclear, some studies have shown that acupuncture increased the μ-opioid receptor binding ability, which could lead patients to demand lower doses of opioids.^[8]^ Acupuncture may also facilitate opioid tapering by reducing adverse events related to opioid use or withdrawal.^[9,10]^ In addition, it may reduce pain intensity and symptoms associated with comorbidities of chronic pain.^[10]^

### 1.4 Why it is important to perform this review

Long-term use of opioids in patients with chronic musculoskeletal pain is known to provide limited pain relief, and the potential side effects may lead to opioid-related death from addiction and overdose. Non-pharmacologic interventions, such as acupuncture have been proposed as alternatives to cope with these problems, address chronic pain,^[6]^ and avoid medication dependence.^[11]^ Although one Cochrane review focused on interventions for the reduction of prescribed opioid use, only 1 acupuncture study was included due to a limited search that did not include Korean or Chinese databases.^[3]^ A systematic review focusing on acupuncture was recently published, but it evaluated only clinical symptoms related to opioid use or withdrawal, not the reduction of prescribed opioids.^[7]^ Therefore, a review with an up-to-date systematic search is needed to evaluate acupuncture for the reduction of opioid consumption; this will help us determine whether acupuncture is an effective treatment option for opioid taper support programs.

### 1.5 Objective

The objective of this systematic review is to evaluate the benefits and harms of acupuncture treatment for the reduction of opioid consumption in patients with chronic pain in comparison to no treatment, sham acupuncture, or other therapies such as pharmacological, physiological, educational, or psychological interventions.

## 2 Methods

### 2.1 Study registration

The protocol of this review has been prospectively registered (CRD42019143486; http://www.crd.york.ac.uk/ PROSPERO) on October 2019. If protocol amendments occur, we will update the changes in the PROSPERO and disclose them in the publications for the results of this study.

### 2.2 Inclusion criteria

#### 2.2.1 Types of studies

Prospective randomized controlled trials (RCTs) of acupuncture treatment for the reduction of opioid use will be included in the review. Non-randomized controlled trials, observational studies, qualitative studies, and laboratory studies will be excluded. Language will not be a restriction for study eligibility.

#### 2.2.2 Types of participants

Data from all adult patients taking opioid medication for non-cancer pain for more than 2 months will be included; there will be no dose limitation. The diagnosis criteria and classification of pain conditions will not be limited and will include various musculoskeletal, mono- or poly-neuropathic, visceral, or head pain.

#### 2.2.3 Types of interventions

Acupuncture treatment is defined in 2 components:

(1) needling with various types of stimulation (eg, manual, electrical, or chemical)
(2) specific points (eg, traditional acupuncture points, myofascial trigger points, or tender points).

Trials examining acupuncture treatment involving the above 2 components will be included in this review. However, trials including non-penetrating stimulation (eg, acupressure, moxibustion, transcutaneous electrical nerve stimulation, or laser therapy) will not be included.

The control will be considered as no treatment, sham acupuncture, or other opioid taper support programs including pharmacological, physiological, educational, or psychological interventions.^[12]^ Only trials in which acupuncture treatment was compared with other forms of acupuncture methods will be excluded. If the acupuncture group simultaneously received acupuncture and other multidisciplinary treatments for pain management or opioid tapering, only trials in which the same program was administered to the both groups will be included.

#### 2.2.4 Types of outcome measures

Primary outcomes

1. Opioid dose (prescribed or consumed): If possible, all opioid doses will be converted into morphine milligram equivalents.
2. Withdrawal symptoms related to opioid reduction

Secondary outcomes

1. Pain intensity
2. Psychological measurements
3. Physical measurements
4. Quality of life (QoL)
5. Clinical global improvement in symptoms
6. Retention of treatments
7. Adverse events related to acupuncture treatment

### 2.3 Search methods for identification of studies

#### 2.3.1 Electronic searches

The following 12 databases will be searched from inception to November 2019: MEDLINE (1946 to November 2019), EMBASE (1980 to November 2019), the Cochrane Central Register of Controlled Trials (*The Cochrane Library*, 2019, Issue 11), the Cumulative Index to Nursing and Allied Health Literature (CINAHL, 1982 to November 2019), the Allied and Complementary Medicine Database (AMED, 1985 to November 2019), the China National Knowledge Infrastructure (CNKI, a Chinese database), the Japan Science and Technology Information Aggregator Electronic (J-STAGE, a Japanese database), and five Korean databases (KoreaMed, Research Information Service System [RISS], Korean Studies Information Service System [KISS], Database Periodical Information Academic [DBpia], and Oriental Medicine Advanced Searching Integrated System [OASIS]).

#### 2.3.2 Search for other resources

The World Health Organization International Clinical Trials Registry Platform will also be checked for ongoing and recently completed trials. Reference lists in the relevant publications will be manually checked for additional eligible trials.

#### 2.3.3 Search strategy

The search terms will consist of three parts: chronic pain (eg, pain, neuropathy, arthritis, fibromyalgia, or headache), opioid use (eg, opioid, morphine, meperidine, or codeine), and acupuncture (eg, acupuncture, electroacupuncture, or dry needling). The special search strategies for MEDLINE are presented online in Appendix 1.

### 2.4 Data collection and analysis

#### 2.4.1 Selection of studies

Two researchers (SL and DHJ) will independently screen potentially eligible articles by reading the titles and abstracts. The researchers will independently select and check their decisions according to predefined criteria on a standard eligibility form. Any disagreement regarding the selection of article will be resolved through discussion, and if a disagreement remains, a third researcher will resolve the disagreement. The flow process will be summarized in a Preferred Reporting Items for Systematic Reviews and Meta-Analysis (PRISMA)-compliant flow chart (http://www.prisma-statement.org).

#### 2.4.2 Data extraction and management

Two researchers (SL and DHJ) will independently read the full text of the selected articles and extract data using a standard data extraction form. This data will include author, year of publication, country, study design, sample size, participants, condition, acupuncture intervention, control intervention, outcome measures, main results, and adverse events. Discrepancies regarding the extracted data will be resolved through discussion or consultation between the researchers. When data in an article is insufficient or unclear, the researchers will contact the first or corresponding author via email to request additional information.

#### 2.4.3 Assessment of risk of bias

Two researchers (SL and DHJ) will independently conduct the quality assessment using the tool for assessing risk of bias based on the Cochrane Handbook for Systematic Reviews of Interventions. The following seven domains will be assessed:

(1) random sequence generation;
(2) allocation concealment;
(3) blinding of participants;
(4) blinding of outcome assessors;
(5) incomplete outcome data;
(6) selective outcome reporting; and
(7) other sources of bias (including factors likely to influence the results, such as extreme baseline imbalance of age, opioid dose, comorbidities, or physical conditions).

The risk of bias will be categorized into 3 levels: low, high, and unclear risk of bias. Any discrepancies in the assessment will be resolved through discussion between the researchers.

#### 2.4.4 Measures of treatment effect

For continuous data, the mean difference (MD) will be presented with 95% confidence intervals (CIs). If the scales measuring the continuous outcomes among the studies in the analysis do not correspond, the standardized mean difference (SMD) will be used. For dichotomous data, the risk ratio (RR) with 95% CIs will be used to measure the treatment effect. Ordinal data will be converted to dichotomous data if the data needs to be pooled. For example, assessments of global improvement graded as ‘recovery’, ‘markedly effective’ ‘effective’, and ‘ineffective’ will be dichotomized into ‘improved’ or ‘not improved’. A patient who achieves more than 50% reduction in opioid consumption will be defined as ‘responder’, and their data will be analyzed as dichotomous data.

#### 2.4.5 Unit of analysis issues

When an outcome variable is assessed repeatedly (at more than 2 follow-up time points after post-treatment), only the last assessment will be chosen for the main analysis.

#### 2.4.6 Dealing with missing data

For missing or incomplete data, we will attempt to contact the first or corresponding authors to request the additional data. If this is not possible, only the available data will be analyzed, and the potential impact of the missing data will be addressed in the discussion.

#### 2.4.7 Assessment of heterogeneity

First, heterogeneity will be judged by visual inspection of the forest plot; then, a chi-square test with a significance level of *P* < .10 will be used to determine the presentation of heterogeneity. The *I*^2^ statistic will also be used to summarize heterogeneity among the trials; *I*^2^ of 0% to 40% might not be important, 30% to 60% may represent moderate heterogeneity, 50% to 90% may represent substantial heterogeneity, and 75% to 100% may represent considerable heterogeneity.^[13]^

#### 2.4.8 Assessment of reporting biases

When more than 10 trials are available, a funnel plot will be used to detect reporting bias.^[13]^ If asymmetry is identified in the funnel plot, Egger linear regression method will be performed.^[14]^

#### 2.4.9 Data synthesis

The meta-analysis will be performed using Review Manager software (RevMan, version 5.3 for Windows; the Nordic Cochrane Centre, Copenhagen, Denmark). A random effects model will be planned to pool data, as considerable clinical heterogeneity involving conditions, acupuncture regimens, and control interventions is expected in the studies included in the review. However, the data will not be pooled when considerable heterogeneity is detected without explanation for the clinical and methodological diversity.^[13]^ When a trial includes more than two acupuncture treatments with different stimulation methods (eg, manual and electrical stimulation) or points (eg, local and distal points), meta-analysis could be performed carefully with consideration of whether the data of different acupuncture treatments could be combined into 1 merged acupuncture treatment.^[15]^ On the contrary, when a trial has more than 2 control interventions (eg, sham acupuncture and another educational program), the data of the acupuncture treatment will be split equally and compared with each control intervention separately.

#### 2.4.10 Subgroup analysis and investigation of heterogeneity

When sufficient data are available, subgroup analysis will be performed according to the following:

1. Style of acupuncture stimulation (eg, manual versus electrical stimulation)
2. Type of control intervention (eg, no treatment, sham acupuncture, or other active therapies)

#### 2.4.11 Sensitivity analysis

When appropriate, sensitivity analysis will be performed to identify whether the results of the review are robust after removing the trials with high risk of bias.

#### 2.4.12 Summary of evidence

The main outcomes (primary outcomes and adverse events) will be summarized in the “Summary of findings” tables. The quality of evidence in the main outcomes will be assessed using the Grading of Recommendations Assessment, Development and Evaluation (GRADE) approach (study limitations, consistency of effect, imprecision, indirectness, and publication bias) with GRADE software. The quality of evidence will be categorized using the following four levels: high, moderate, low, and very low quality.^[13]^

### 2.5 Ethics and dissemination

As this is a systematic review, ethical approval and informed consent were not necessary. The results of this review will be disseminated through conference presentations and peer-reviewed journal articles.

## 3 Discussion

This is the study protocol of a systematic review and meta-analysis on the use of acupuncture treatment for reduction of opioid consumption in patients with chronic pain.

Many studies have shown that perioperative acupuncture treatment effectively reduces acute opioid consumption after surgery.^[16,17]^ Furthermore, a recent systematic review showed that conventional acupuncture or transcutaneous electric acupoint stimulation reduced consumption of opioid analgesics, especially on the first day after surgery.^[18]^ Though, in the last several years, the long-term abuse of opioid therapy has been coined an opioid crisis and recognized as one of the most challenging problems of public health, research for reducing long-term opioid consumption is not well known. Recently, various federal regulatory and professional organizations have started to encourage the use of multidisciplinary approaches, including nonpharmacologic interventions, to reduce long-term opioid consumption and opioid use disorder.^[19]^ In this context, acupuncture treatment, which is already supported by rigorous evidence for chronic pain, has received attention as the most promising treatment option for this strategy.^[20,21]^ Zheng and colleagues presented the possibility of using electroacupuncture to reduce opioid consumption in patients with chronic musculoskeletal pain through conducting sham-controlled clinical trials.^[9,10]^ However, to determine the role of acupuncture in the multidisciplinary approach used in opioid taper support programs, there must be comprehensive evidence of acupuncture for the reduction of opioid consumption.

Therefore, we will conduct a systematic review searching all relevant literature, without any language restrictions, in the Korean, Chinese, and Japanese databases to include any relevant trials of acupuncture for the reduction of opioid consumption in patients with chronic pain. The results of this systematic review will be useful for patients, practitioners, and health policy-makers who wish to include acupuncture among the multidisciplinary treatment options available in opioid taper support programs. Moreover, this review will be constructive for researchers planning further clinical trials.

## Acknowledgments

The author would like to thank Professor Kun Hyung Kim and Jung Won Kang for encouraging suggestions and helpful comments.

## Author contributions

**Conceptualization:** Seunghoon Lee

**Funding acquisition:** Seunghoon Lee

**Methodology and project administration:** Seunghoon Lee

**Writing (original draft):** Seunghoon Lee

**Writing (review and editing):** Dae-Hyun Jo, Seunghoon Lee

Seunghoon Lee orcid: 0000-0003-0088-2296.

## Supplementary Material

Supplemental Digital Content
