# Supplementary material for: Acupuncture for reduction of opioid consumption in chronic pain: A systematic review and meta-analysis protocol
Source: Medicine (Baltimore). 2019 Dec 20;98(51):e18237. doi: 10.1097/MD.0000000000018237 (PMC6940175; doi:10.1097/MD.0000000000018237)
Supplement: Supplemental Digital Content [file medi-98-e18237-s001.docx]

**Appendix 1. Search strategies (*MEDLINE (Ovid Online))***

1. exp Chronic Pain/
2. exp Pain, Intractable/
3. exp Fibromyalgia/
4. exp Neuralgia/
5. exp Headache Disorders/
6. exp Arthritis/
7. (pain* or fibromyalgia* or neuralgia* or neuropath* or headache* or migraine* or arthriti* or osteoarthriti*).mp.
8. 1 or 2 or 3 or 4 or 5 or 6 or 7
9. exp Analgesics, Opioid/
10. exp Opiate Alkaloids/
11. exp Morphine Dependence/
12. (opioid* or opiate* or morphine or meperidine or methadone or buprenorphine or fentanyl or hydrocodone or oxycodone or codeine).mp.
13. 9 or 10 or 11 or 12
14. exp Acupuncture/
15. exp Acupuncture therapy/
16. exp Electroacupuncture/
17. exp Acupuncture points/
18. exp Meridians/
19. (acupuncture or electroacupuncture or electro-acupuncture or auriculoacupuncture or auriculo-acupuncture or dry needling or acupuncturist* or acupoint* or meridian*).mp.
20. 14 or 15 or 16 or 17 or 18 or 19
21. 7 and 12 and 19
